# Portable and low-cost hologram verification module using a snapshot-based hyperspectral imaging algorithm

**DOI:** 10.1038/s41598-022-22424-5

**Published:** 2022-11-02

**Authors:** Arvind Mukundan, Yu-Ming Tsao, Fen-Chi Lin, Hsiang-Chen Wang

**Affiliations:** 1 Department of Mechanical Engineering, Advanced Institute of Manufacturing With High Tech Innovations (AIM-HI) and Center for Innovative Research On Aging Society (CIRAS), National Chung Cheng University, 168, University Rd., Min Hsiung, Chia Yi, 62102 Taiwan; 2 Department of Ophthalmology, Kaohsiung Armed Forces General Hospital, 2, Zhongzheng 1st. Rd., Lingya District, Kaohsiung City, 80284 Taiwan

**Keywords:** Engineering, Optics and photonics

## Abstract

One of the challenges in differentiating a duplicate hologram from an original one is reflectivity. A slight change in lighting condition will completely change the reflection pattern exhibited by a hologram, and consequently, a standardized duplicate hologram detector has not yet been created. In this study, a portable and low-cost snapshot hyperspectral imaging (HSI) algorithm-based housing module for differentiating between original and duplicate holograms was proposed. The module consisted of a Raspberry Pi 4 processor, a Raspberry Pi camera, a display, and a light-emitting diode lighting system with a dimmer. A visible HSI algorithm that could convert an RGB image captured by the Raspberry Pi camera into a hyperspectral image was established. A specific region of interest was selected from the spectral image and mean gray value (MGV) and reflectivity were measured. Results suggested that shorter wavelengths are the most suitable for differentiating holograms when using MGV as the parameter for classification, while longer wavelengths are the most suitable when using reflectivity. The key features of this design include low cost, simplicity, lack of moving parts, and no requirement for an additional decoding key.

## Introduction

Various optical systems, such as spectral imaging^1–3^, optical coherence tomography (OCT)^4^, photoacoustic (PA) imaging mechanism^5^, laser scanning micro-profilometry^6^, ultraviolet–visible (UV–Vis) spectroscopy^7^, optical fiber sensor^8^, and scanning microscopy^9^, are available. In OCT, low coherence light is employed to obtain 3D images from within optical scattering media^10,11^. The PA imaging technique uses optical illumination and ultrasound wave detection to visualize optical absorption, which is frequently related to the properties of an object^12,13^. In UV–Vis spectroscopy, the absorption or reflection properties of a material are compared in the ultraviolet and visible bands of the electromagnetic spectrum^14,15^. In scanning microscopy, accelerated electrons are used as the source of light^16,17^. Most of these optical systems have been employed to detect different types of fraud, including counterfeit currencies, pharmaceutical drugs, documents, and artwork. However, one application in which optical systems are not widely used is duplicate hologram detection and classification. Holograms, also known as diffractive optically variable image devices, are optically variable objects^18,19^. They change appearance when viewed from a different angle or under a different lighting system. Therefore, designing an optical system for detecting and classifying any holograms is challenging and expensive^20^. Moreover, the portability of an optical system should be considered in this case. One of the methods that can overcome all the aforementioned challenges in the classification of holograms is hyperspectral imaging (HSI). HSI is an evolving pioneering statistical and heuristic spectrometric technology^21,22^. It is a nondestructive technique that is widely used to examine a broad spectrum of light rather than merely examining the primary colors, i.e., red, green, and blue (RGB) in the pixels of an image^23,24^. HSI has been used in various field and applications, such as cancer detection^25^, air pollution monitoring^26^, remote sensing^27^, agriculture^28^, astronomy^29^, quality control^30^, environment monitoring^31^, and vegetable classification^32^. In an HSI image, every pixel not only contains the primary colors but also the absorption and reflectance data^33,34^. Each pixel includes spectral information, forming 3D values on 2D images^35^. This phenomenon is known as the hyperspectral data cube^22^. HSI data are assumed to be sampled spectrally at more than 20 equally distributed wavelengths. HSI can also be extended beyond the visible spectrum (VIS)^36^ and into near-infrared^37^ and far-infrared^38^ spectra.

At present, nearly all HSI applications are restricted to research laboratories because these instruments are heavy, expensive, and laborious to use^35^. Sumriddetchkajorn and Intaravanne demonstrated the ability of HSI to verify the authenticity of a credit card by analyzing the hologram printed on it^1^. They obtained different color spectra of the hologram in the credit card by using white light at different angles. However, the instrument they designed is expensive and not portable. Another study built a portable cylindrical light module that can be attached to a mobile device to verify holograms in currency notes^39^. Although this instrument is portable, it can verify only a small hologram. Moreover, the quality of the camera will vary among different phone models used to acquire the image, and this condition can considerably affect the analysis results. The major goal of HSI research is to make HSI more affordable, user-friendly, and compact.

Therefore, the current study proposes and demonstrates a portable and low-cost HSI-based housing module to differentiate between original and duplicate holograms. The system consists of a Raspberry Pi 4 processor, a Raspberry Pi version 2 camera module, a thin film transistor (TFT) display, a light-emitting diode (LED) lighting system, and a LED Dimmer. A VIS-HSI technology is also developed to simulate the RGB values captured by the camera into a hyperspectral image. A region of interest (ROI) is selected, and the mean gray value (MGV) acquired from four original holograms is used as a reference and compared with that obtained from four duplicate holograms. A 98% confidence interval (CI) is formed around the MGV of every wavelength and used as a classification criterion. The results demonstrate that the four duplicate holograms are easily differentiated from the original ones. Moreover, the reflectance data of the ROI are measured, indicating that the original and duplicate holograms have different reflective patterns in the longer wavelengths.

## Results and discussion

**Figure 1:**
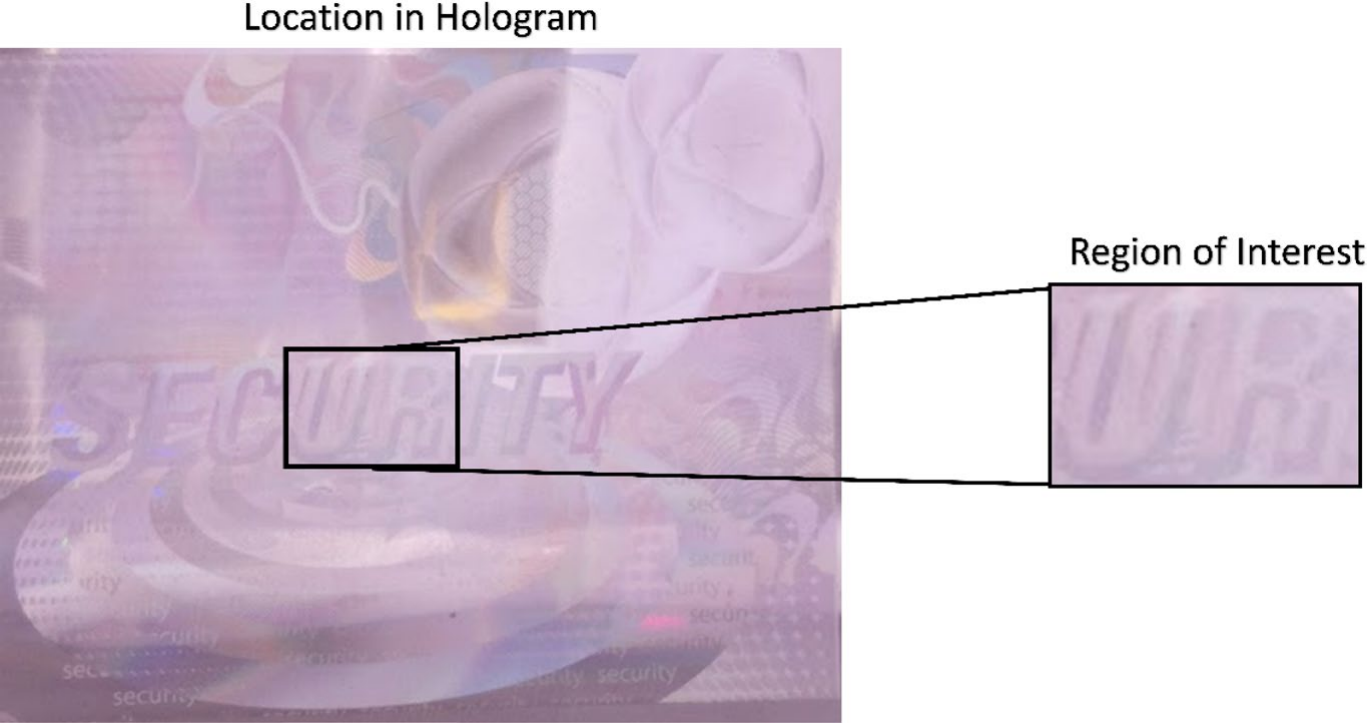
Location of the ROI in the hologram.

**Figure 2:**
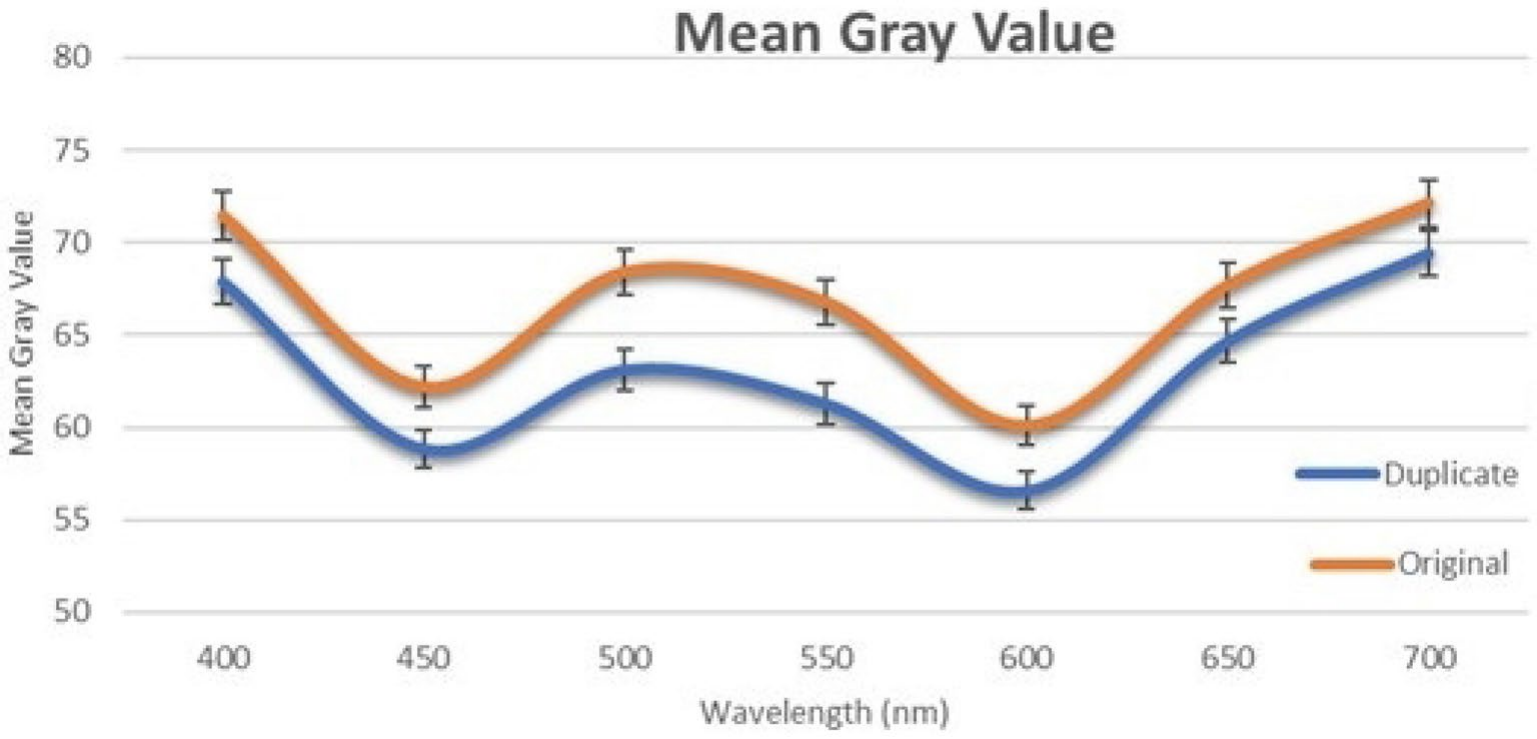
Mean MGV of the duplicate and original samples. The error bar represents a 98% CI range in the specified wavelength (n = 8).

In this study, a Python-based Windows application is developed to capture the real-time image from the Raspberry Pi camera. One specific ROI is extracted from the image that is converted into a hyperspectral image. Finally, the hologram is classified either as an original or a duplicate based on MGV. The ROI consists of the fourth and fifth letters in the word “SECURITY,” i.e., “UR.” This region is selected because it is placed at the center of the hologram, and thus, easily accessible, as shown in Fig. 1. This ROI has a height and width of 0.6 mm and 0.9 mm, respectively. It has a total of 33,810 pixels. This research is a pilot study and hence only four duplicate and four original holograms are used as a reference for numerical analysis provided by K Laser Technology Inc. For the ROI, the MGV is measured in the VIS-HSI band between 400 and 700 nm. MGV is the average measure of the brightness of all the pixels in the image^40,41^. In a color image, the gray value can have values between 0 and 255. In the past, many studies used MGVs for image classification and detection, because it is one of the reliable classification methods^40,42–44^. Figure 2 shows the mean of the duplicate and original samples between 400 and 700 nm (see Supplement 1 Sect. 3 for the detailed plot of all the samples).

**Figure 3:**
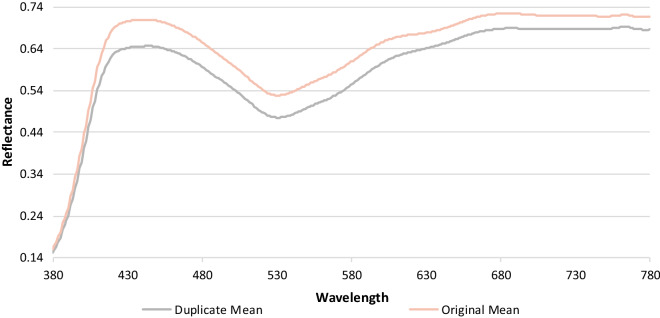
Mean of the reflectance spectra of four duplicate and four original samples.

The original and duplicate holograms can be easily differentiated. The RMSE of the MGVs in the shorter wavelength between 400 and 500 nm is 3.4664, while that in the longer wavelength between 600 and 700 nm is 3.1126. However, the RMSE of MGVs in the middle wavelengths is 5.4049. Therefore, based on RMSE, we can infer that the middle wavelengths between 500 and 600 nm are the most suitable for the classification of holograms (see Supplement 1 Sect. 2 for entropy measurement). The samples are classified into two classes: original and duplicate holograms. As shown in Fig. 3, a 98% CI is also calculated around the average MGVs of both classes for each wavelength. CI represents the range in that specific wavelength wherein the MGV of a sample belonging to that specific class will probably fall. Although 95% is the most commonly used CI, 98% CI can be used because the MGVs of the samples fall within a narrow range in this study. On this basis, the hologram will be classified as original if the MGV falls within its class; otherwise, the hologram will be classified as duplicate. Although, the developed method can measure the MGVs from 400 till 780 nm, the MGVs become similar for the duplicate and the original holograms after 700 nm falling under the 98% CI. Hence, in this study the MGVs between 400 and 700 nm were only considered.

The reflectance of the center pixel of all the samples in this study is also calculated using the VIS-HSI algorithm developed in this study. Figure 3 shows the mean reflectance spectrum of the duplicate and original holograms in the visible spectrum (see Supplement 1 Section 3 for individual plots for every sample). In the longer wavelengths, specifically after 580 nm, a clear difference exists between the original and duplicate samples in the reflectance. This result is consistent with those of previous image classification studies that used HSI^45–47^. Classifying images is easier in the long wavelengths than in the middle or short wavelengths in HSI. Lim and Murukeshan calculated the reflectance spectra of original and duplicate banknotes^48^. A difference between the reflectance of the original and duplicate banknotes was only observed in longer wavelengths above 550 nm. Qin et al. measured the reflectance spectra of grapefruit samples with normal and different diseased skin conditions^49^. A clear difference existed in the reflectance data only between wavelengths 600 nm and 800 nm, which are in the longer wavelength region. Both the MGVs and the reflectance values can be used to classify the hologram, MGV has been specifically used to classify because the RMSE value in the wavelengths 500 and 550 are greatest in MGV. Therefore, a better classification performance can be achieved by increasing the CI to 90%.

In this study, a stand-alone Python-based Windows application is also developed to classify holograms. To capture images, the Raspberry Pi web camera interface is installed in the Raspberry Pi operating system. The live feed of the Raspberry Pi camera can be directly accessed by the Windows application by clicking the “Start” button, and the camera can also be turned off by clicking the “Stop” button. The “Capture” button is used to capture the current image that will be used for classification. In the application, the frames per second and frame number are displayed. The user must input parameters, such as the wavelength of the narrowband in which the sample will be analyzed and the gain value specific to each narrowband by clicking the “Ok” button. Once the “Analyze” button is clicked, the application will convert the RGB image captured by the Raspberry Pi camera into a hyperspectral image and crop the ROI from the image. Then, the MGV of the sample will be calculated and classified into its respective class based on the narrowband wavelength inputted by the user. The final result will be displayed at the bottom of the application either as “The Hologram is Original” or “The Hologram is Counterfeit,” (to see the application built in this study refer supplement 1 Figure S10).

## Methods

**Figure 4:**
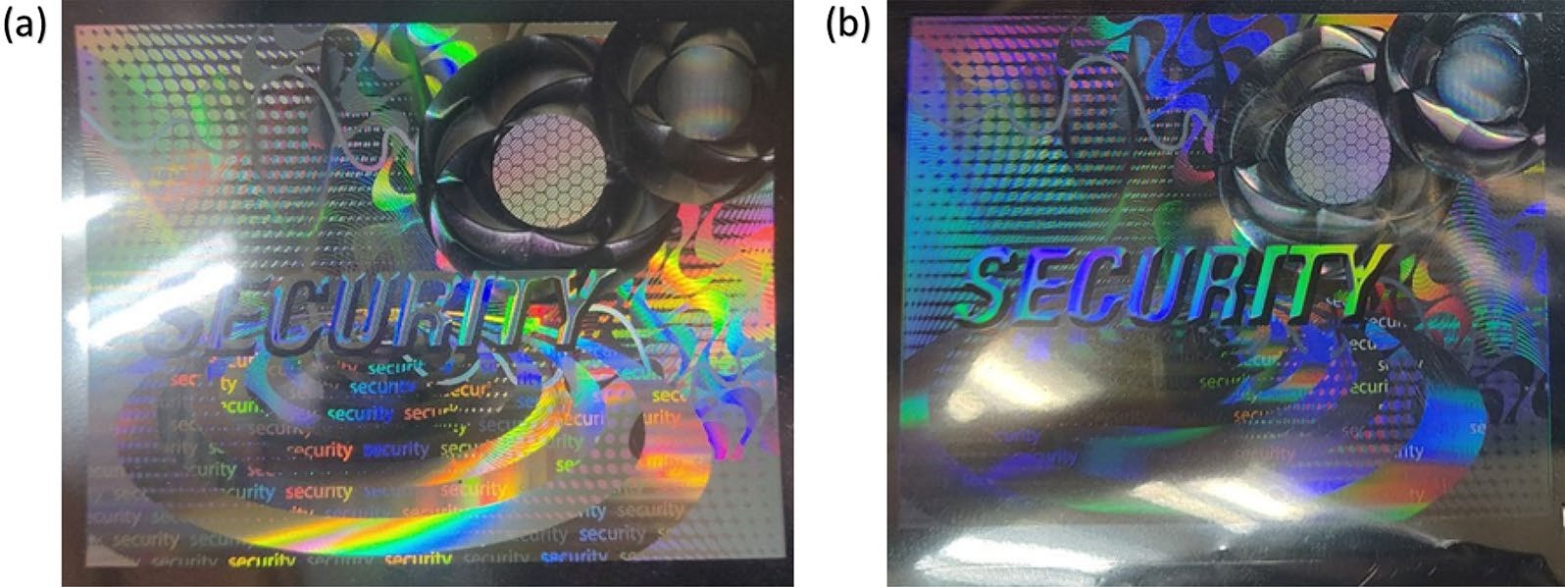
(**a**) Original hologram and (**b**) duplicate hologram.

One of the challenges in differentiating between duplicate and original holograms is reflection. A minor change in lighting angle will create an entirely different reflection pattern. Holograms can be differentiated by capturing and analyzing the reflection data from different incident angles. However, the reflection pattern will vary under different light sources. Therefore, the light source should also be constant. As shown in Fig. 4, a slight change in lighting conditions can lead to a drastic change in the reflection from the final image. Hence, a module must be designed to reduce external light, and lighting conditions must be the same for all holograms.

**Figure 5:**
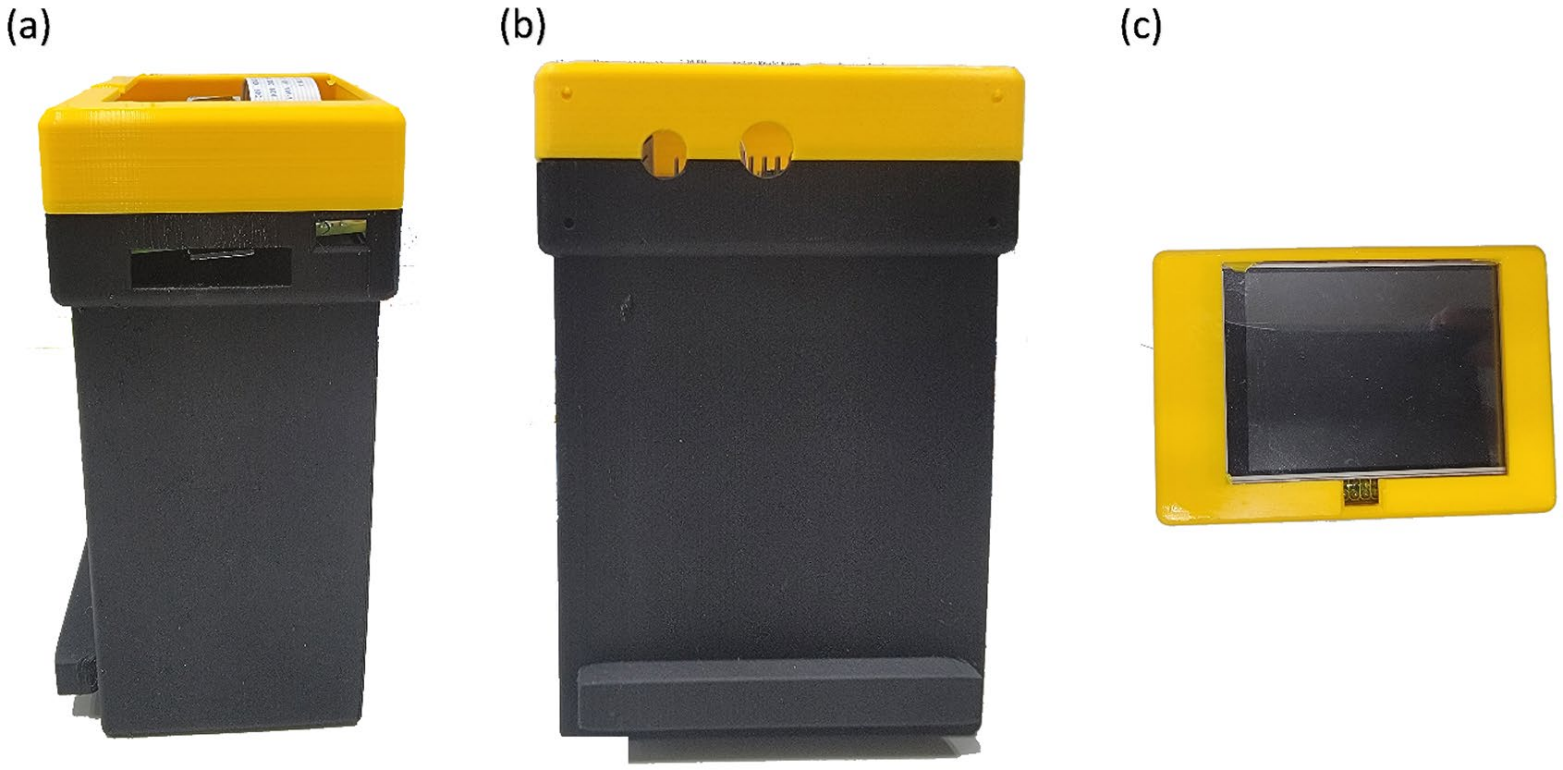
(**a**) Side view, (**b**) front view, and (**c**) top view of the 3D printed design.

A module that could accommodate all the electronic components and reduce external light noises was designed in the current study. The design was 3D printed using Ultimaker Cura 3. The final product is presented in Fig. 5. The critical factors considered in this study were minimizing the number of optoelectronic components, reducing the size of the design, and optimizing the positioning of different components. In any image processing method based on hyperspectral or multispectral studies, a spectrometer, an optical head, or a multispectral LED board is typically used, making the module costly and fixed. Hence, the number of components must be reduced to build a low-cost and compact module. Accordingly, only a minimum number of components are utilized in the current study. The whole module can be divided into two units: the processing system and the optical system. The processing unit consists of three components: a Raspberry Pi 4 microprocessor, a Raspberry Pi camera module version 2, and a TFT display unit. Meanwhile, the optical system consists of a LED strip, a diffuser, and a LED dimmer (for the detailed specifications of these components, refer to Supplement 1 Section 1).

**Figure 6:**
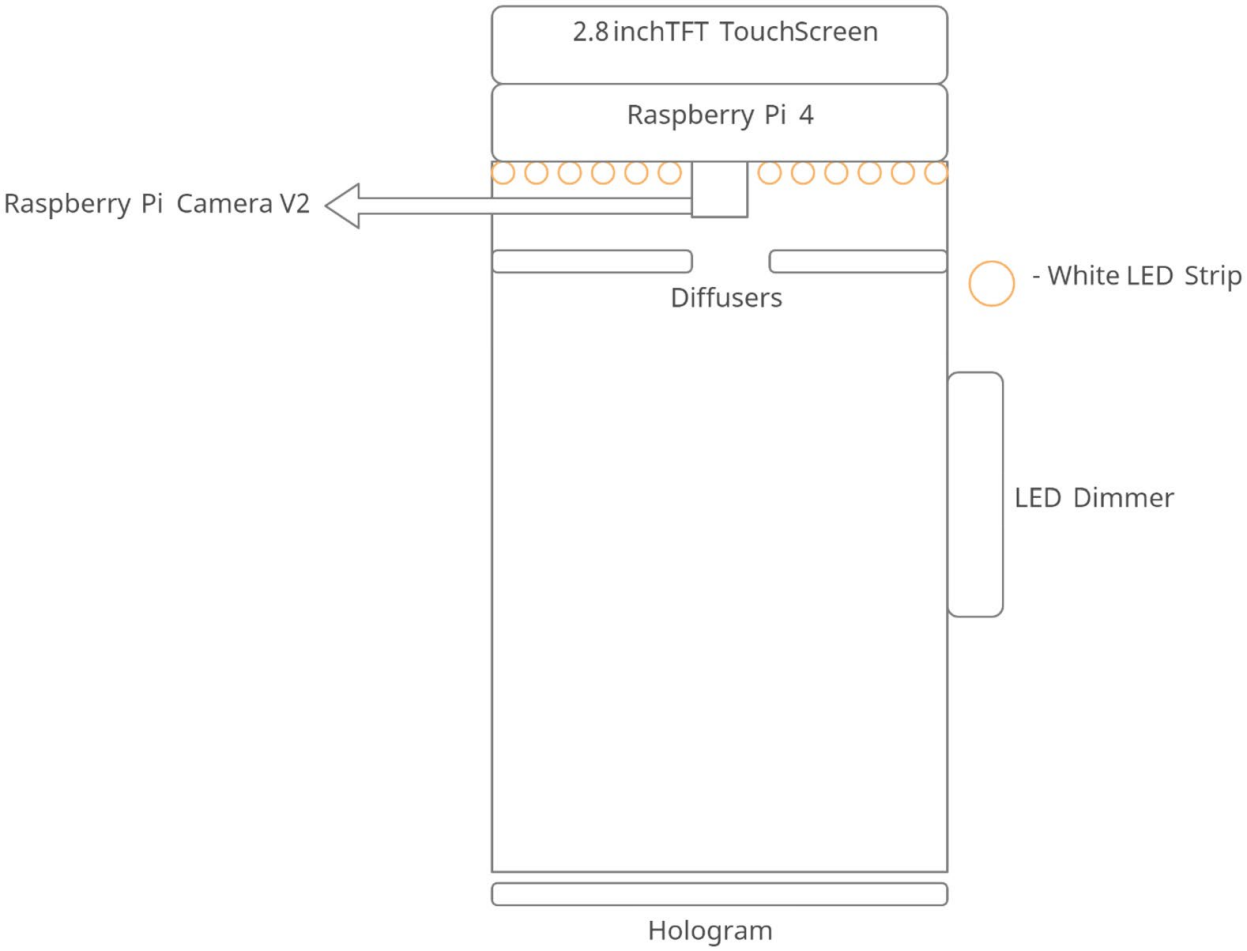
Schematic of the imaging system.

The schematic of the imaging system is presented in Fig. 6. The Raspberry Pi 4 computer Model B is used for processing, while the Raspberry Pi camera module version 2 is used to capture the images of a hologram. The processor is connected to an Adafruit Pi TFT 320 × 240 2.8" touch screen to control the processing unit. The optical system consists of a 3000 K chip on board LED strip light, which is fixed onto the design. The LED strip light is connected to a LED dimmer switch to control brightness. The COB LEDs has a uniform spectral response (no cyan dip) across the blue, green, and red color spectrum. However, this lighting system is not even, but rather, a pointed light source. An opal profile diffuser is also used to reduce the transmittance rate and distribute the light source evenly.

### Snapshot-based RGB to HSI conversion algorithm

**Figure 7:**
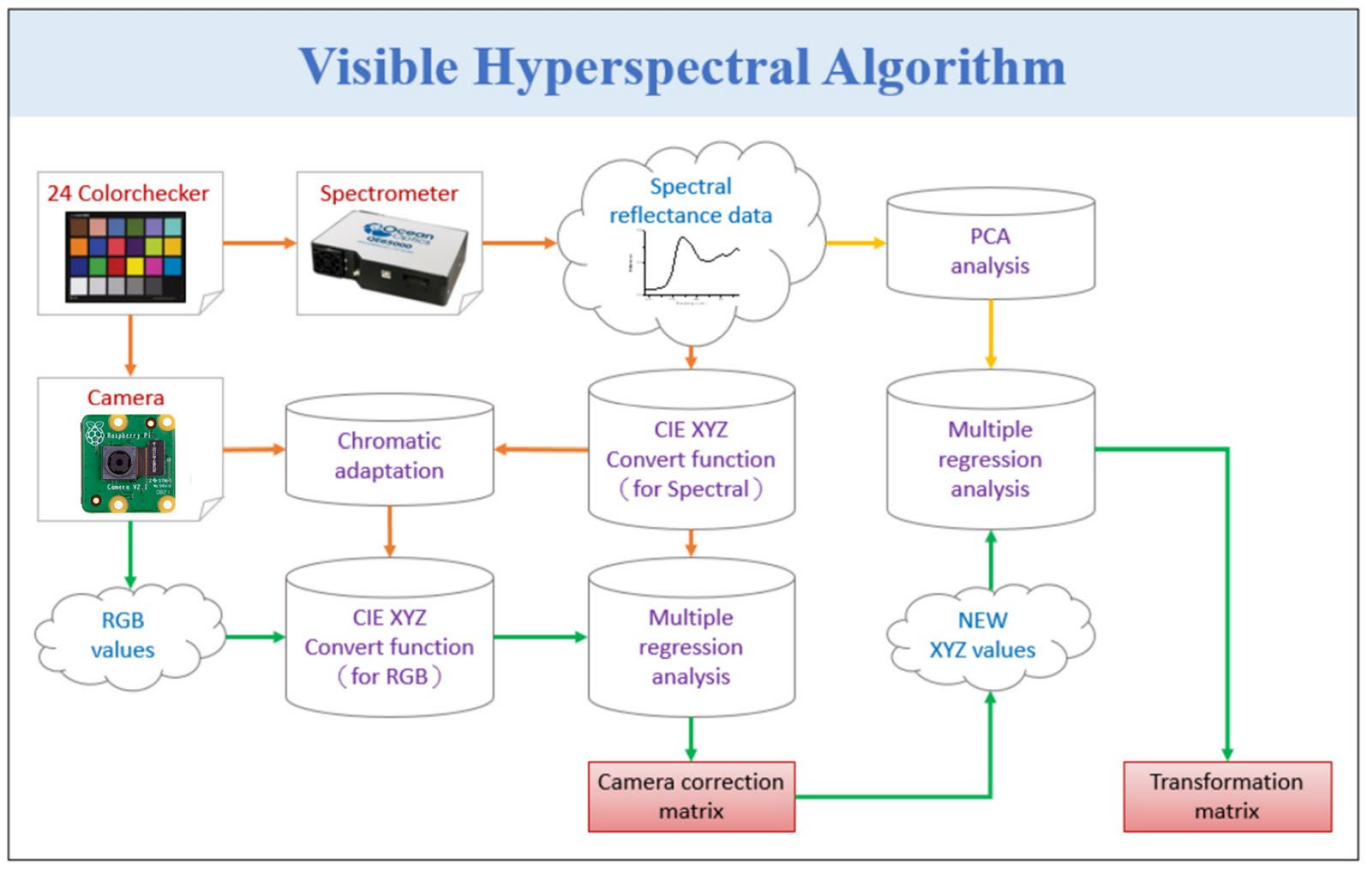
VIS construction process.

The core concept of the VIS-HSI technology is to endow a common digital camera with the function of a spectrometer, i.e., the image captured by the camera contains spectral information. To achieve this, a relationship matrix between the camera and the spectrometer that can be used to construct VIS-HSI technology must be established. The construction process of this technology is illustrated in Fig. 7. First, a camera (Raspberry Pi Camera) and a spectrometer (Ocean Optics, QE65000) is provided with multiple common targets as reference for the analysis. In the current study, a standard 24-color checker (X-Rite classic) is selected as the target, because it contains the most important colors (blue, green, red, and gray) and other common colors found in nature. To correct camera errors because they may be affected by inaccurate white balance, the standard 24-color card must be passed through the camera and the spectrometer to obtain 24-color patch images (sRGB, 8 bit) and 24-color images, respectively. Then, the 24-color patch image and the 24-color patch reflectance spectrum data are converted into CIE 1931 XYZ color space (for the individual conversion formulae, see Supplement 1 Sect. 4).

In the camera, the image (JPEG, 8 bit) is stored by the sRGB color space specification. Before converting an image from the sRGB color space into the XYZ color space, the respective R, G, and B values (0–255) must be converted into a smaller scale range (0–1), and then the sRGB values are converted into linear RGB values through gamma function conversion. Finally, the conversion matrix is used to convert the linear RGB value into the XYZ values normalized in the XYZ gamut space. In the spectrometer, the reflection spectrum data must be converted into the XYZ color gamut space, the XYZ color-matching functions, and the light source spectrum. The Y value of the XYZ color gamut space is proportional to the brightness; hence, the maximum brightness of the light source spectrum is calculated, and the Y value is standardized to 100 to obtain the brightness ratio (k). Finally, the reflection spectrum data are converted into the XYZ value (XYZ_Spectrum_) normalized in the XYZ color gamut space. Multiple regression is performed to obtain the correction coefficient matrix C for calibrating the camera, as shown in Eq. (1). The variable matrix V is obtained by analyzing the factors that may cause errors in the camera, such as nonlinear response, dark current, inaccurate color separation of the color filter, and color shifting.

1 $$\left[\mathrm{C}\right]=\left[{\mathrm{XYZ}}_{\mathrm{Spectrum}}\right]\times \mathrm{pinv}(\left[\mathrm{V}\right])$$

Once the camera is calibrated, the corrected X, Y, and Z (XYZ_Correct_) values can be obtained using Eq. (2). The conversion matrix M is obtained using the spectrometer and the reflection spectrum data (spectrum) of the 24 color blocks. The average root-mean-square error (RMSE) of XYZ_Correct_ and XYZ_Spectrum_ is 0.19, which is negligible in this case. The important principal components are identified by performing the principal component analysis (PCA) on R_Spectrum_. Moreover, multiple regression is performed on the extracted principal component scores, which are combined with the previously obtained results to determine the conversion matrix M.

2 $$\left[{\mathrm{XYZ}}_{\mathrm{Correct}}\right]=\left[C\right]\times [V]$$

To convert XYZ_Correct_ into R_Spectrum_, the dimensions of R_Spectrum_ must be reduced to increase the correlation between each dimension and XYZ_Correct_. Therefore, R_Spectrum_ is analyzed via PCA to obtain the eigenvectors. The six principal components are used in dimensionality reduction because these six groups of principal components have been able to explain 99.64% of data variability. The corresponding principal component score can be used for regression analysis with XYZ_Correct_. In the multivariate regression analysis of XYZ_Correct_ and the score, the variable V_Color_ is selected because it has all the listed possible combinations of X, Y, and Z. The transformation matrix M is obtained using Eq. (3), and then XYZ_Correct_ is passed through Eq. (4) to calculate the analogue spectrum (S_Spectrum_). Finally, the obtained 24-color block analogue spectrum (S_Spectrum_) is compared with the 24-color block reflection spectrum. The RMSE of each color block is calculated, and the average error is 0.056, which is negligible. The average color difference between the 24-color block analogue spectrum and the 24-color block reflection spectrum is 0.75, suggesting that distinguishing color difference is difficult. When the processed reflection spectrum color is reproduced, the color is reproduced accurately. The VIS-HSI technology constructed from the above process can simulate the reflection spectrum from the RGB values captured by the monocular camera to obtain the VIS hyperspectral images.

3 $$\left[M\right]=\left[Score\right]\times pinv(\left[{V}_{Color}\right])$$

4 $${[SSpectrum]}_{380\sim 780nm}=\left[EV\right]\left[M\right][{V}_{Color}]$$

By using this method, an RGB image captured by a digital camera can be converted into an HSI image without using a spectrometer, an optical head, or a hyperspectral camera. By eliminating these components, the machine designed in this study is low cost and highly portable but can still differentiate between duplicate and original holograms.

## Conclusion

In this study, a portable and low-cost module has been designed to capture, classify, and detect duplicate hologram. This specific design reduces the external light noises which will cause uneven reflection pattern and provides an even light distribution throughout the surface of the hologram. Also, a VIS-HSI algorithm has been built to convert the RGB images captured by the Raspberry Pi camera to a hyperspectral image. A region of interest is selected from the hyperspectral image and the mean grey value is measured and 98% confidence interval is calculated in the visible band between 400 and 700 nm. Based on the MGVs the holograms are classified as either original or duplicate. Finally, a stand-alone Python-based Windows application is also built which is used to control the Raspberry Pi micro-processor and access the real-time feed of the Raspberry Pi camera housed in the portable and low-cost module. Based on the user defined narrow band wavelength values, the application will analyse the hologram and display which class does the sample belong to. The future scope of this study is to use same methodology to classify the counterfeit currencies from the original currency. The same design could also be used to design a NIR-HSI conversion algorithm and a low cost NIR-HSI module can be developed.

## Supplementary Information

Supplementary Information.

## Data Availability

The datasets used and/or analyzed during the current study available from the corresponding author on reasonable request.
