# Supplementary material for: Portable and low-cost hologram verification module using a snapshot-based hyperspectral imaging algorithm
Source: Sci Rep. 2022 Nov 2;12:18475. doi: 10.1038/s41598-022-22424-5 (PMC9630442; doi:10.1038/s41598-022-22424-5)
Supplement: Supplementary file 1 — Supplementary Information. [file 41598_2022_22424_MOESM1_ESM.docx]

# **Portable and Low-cost Hologram Verification Module Using a Snapshot-Based Hyperspectral Imaging Algorithm: Supplement Material**

**Arvind Mukundan^1^, Yu-Ming Tsao^1^, Fen-Chi Lin^2,*^, and Hsiang-Chen Wang ^1,*^.**

^1^ Department of Mechanical Engineering, Advanced Institute of Manufacturing with High tech Innovations (AIM-HI) and Center for Innovative Research on Aging Society (CIRAS), National Chung Cheng University, 168, University Rd., Min Hsiung, Chia Yi 62102, Taiwan; d09420003@ccu.edu.tw (A.M), d09420002@ccu.edu.tw (Y.-M.T.).

^2^ Ophthalmology, Kaohsiung Armed Forces General Hospital, 2, Zhongzheng 1st.Rd., Lingya District, Kaohsiung City 80284, Taiwan; eses.taiwan@gmail.com

Corresponding author: Fen-Chi Lin ([eses.taiwan@gmail.com) and](mailto:eses.taiwan@gmail.com)%20and) Hsiang-Chen Wang (hcwang@ccu.edu.tw)

**1. Instrument Specification**

In this study, a total of six instruments have been used including a microprocessor, monocular camera, LED strip, diffuser, LED dimmer, and a TFT touch screen. Table S1 describes all the instruments used. The microprocessor used was Raspberry Pi 3 Model B+ which has 1GB LPDDR2 SDRAM. It was operated using a computer through VLC. The camera used to capture the hologram was Raspberry Pi Camera Module 2 which has a Sony IMX219 8-megapixel sensor. The screen used to control the processor was Adafruit’s 2.8-inch PiTFT – Touchscreen. A 3000K COB light was also used along with a dimmer. An opal white diffuser sheet is also used to diffuse the light evenly onto the hologram.

| Part | Component | Model | Cost | Manufacturer |
| --- | --- | --- | --- | --- |
| Processor | Raspberry Pi V4 | Model B+ | $35.00 | Raspberry Pi |
| Camera | Raspberry Pi Camera | V2 | $25.00 | Raspberry Pi |
| LED and Dimmer | COB LED Strip | COB | $25.00 | PAUTIX |
| Diffuser | Opal Diffuser | - | $5 | Guangzhou co. ltd |
| TFT Screen | 320x240 2.8" TFT | - | $35 | Adafruit |

Table S1. Components used to build the module.

**2. Entropy Measurement**

In this study, the entropy of the three duplicate and three original holograms were selected and the entropy was measured. Entropy in an image can be defined as the measure of the degree of randomness in the image. From Figure S1 and Figure S2 which represents the entropy for the shorter wavelength, the selected region of interest (ROI) is similar. However, in the middle wavelength, the ROI is different thereby making it an id choice to analyze the mean grey value (MGV). In the longer wavelength again the entropy in the ROI is similar. This is the reason why the root mean squared error in the shorter and the longer wavelength is small while higher in the middle wavelength.


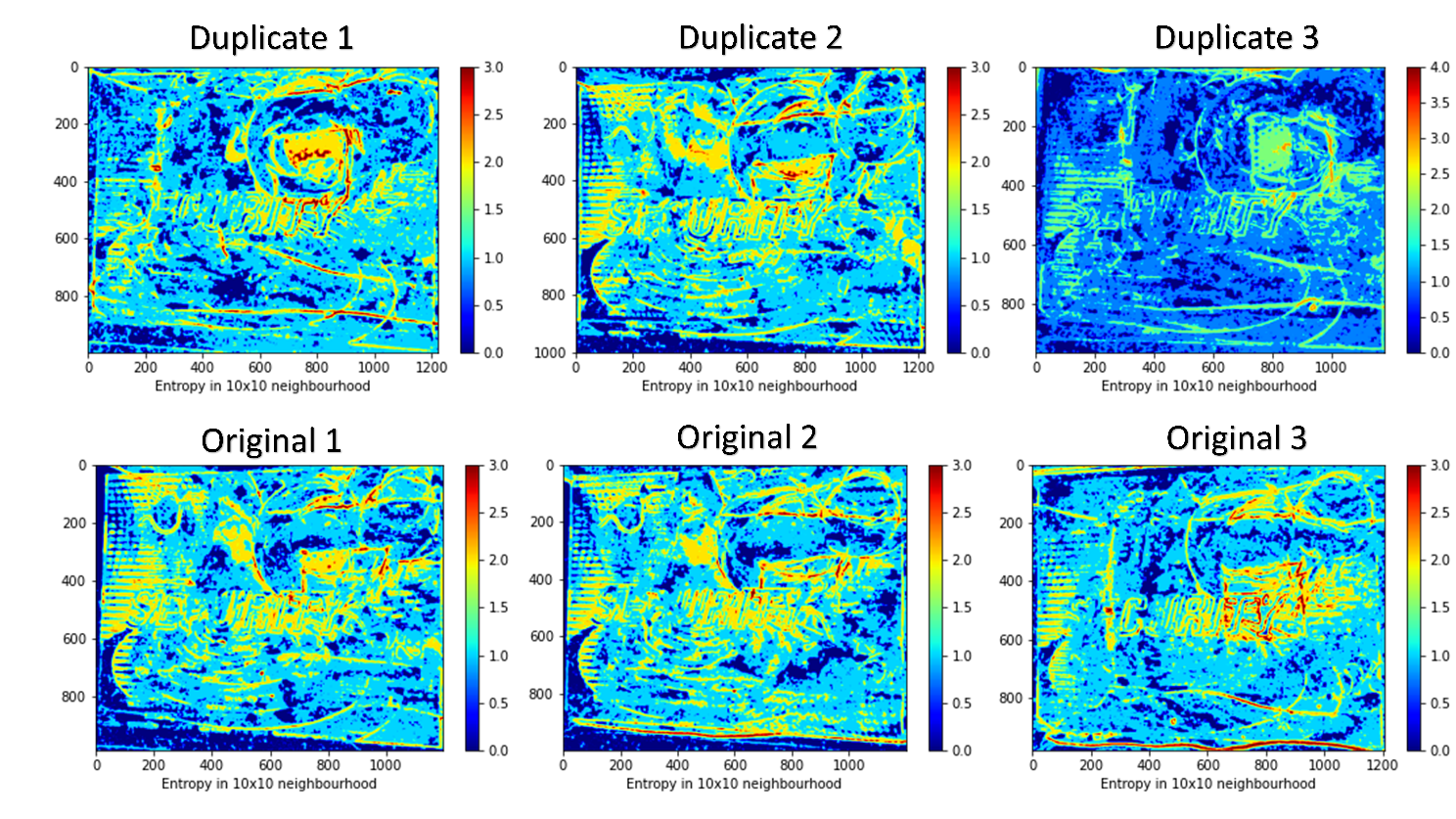


Figure S1. Entropy at 400 nm.


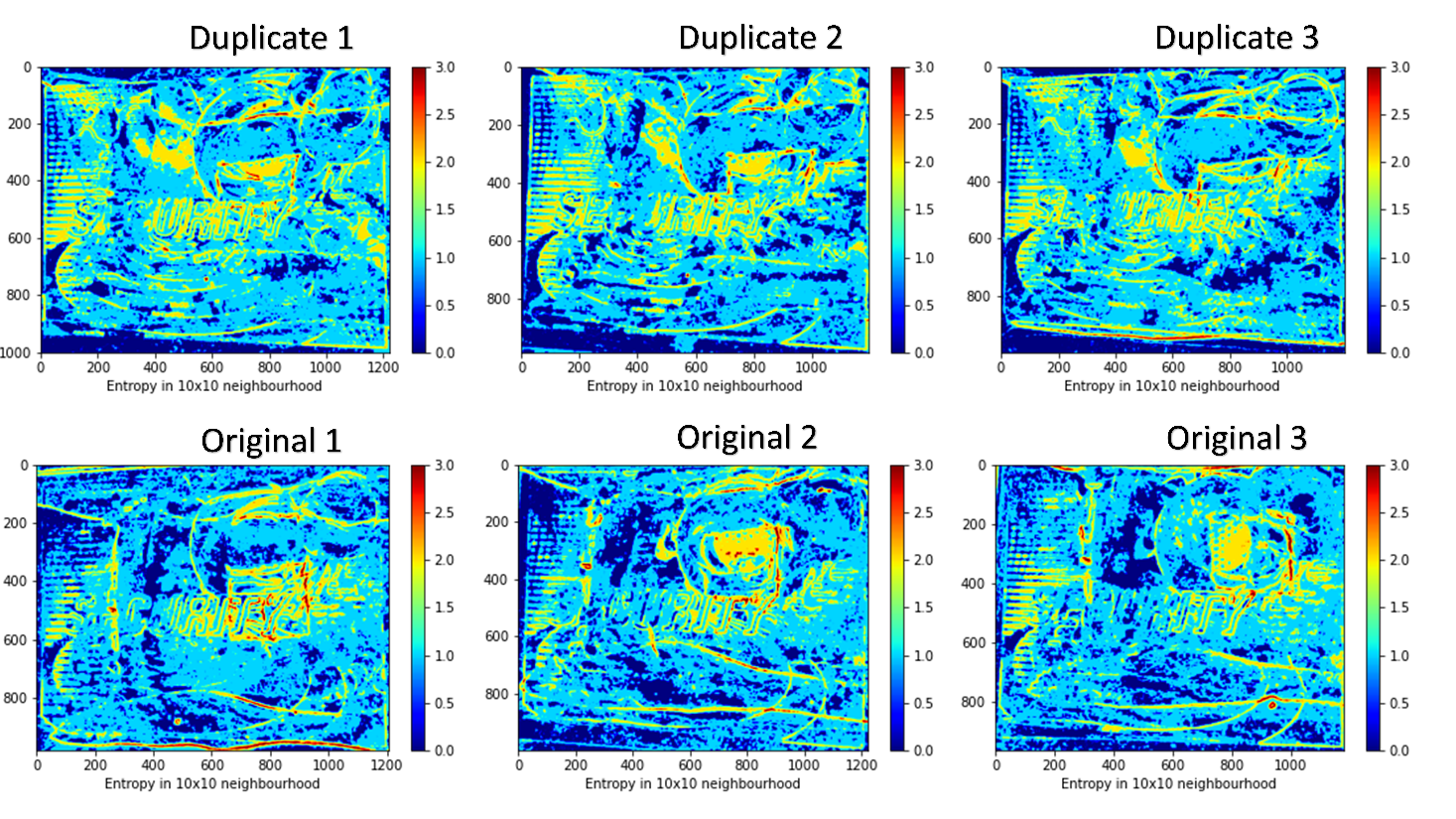


Figure S2. Entropy at 450 nm.


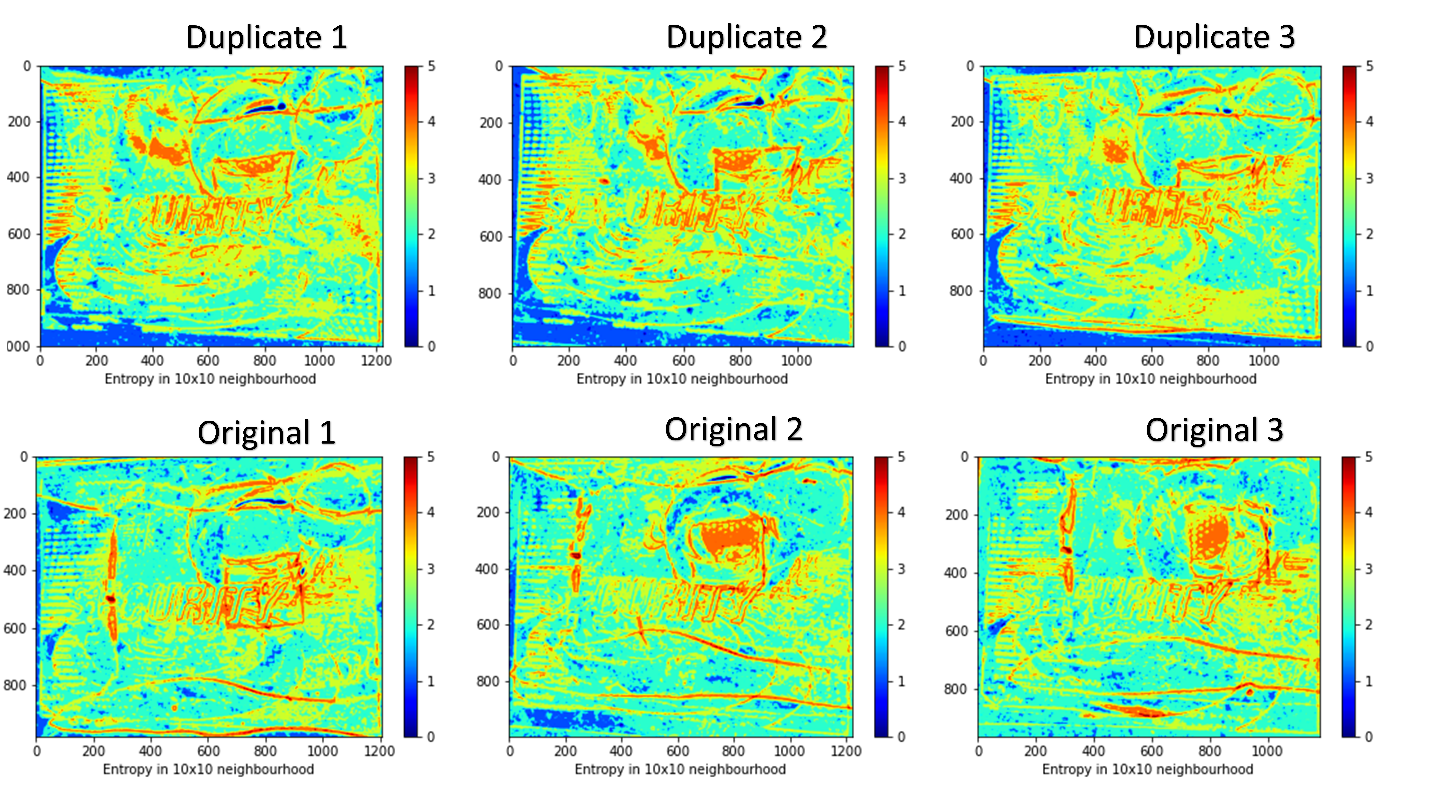


Figure S3. Entropy at 500 nm.
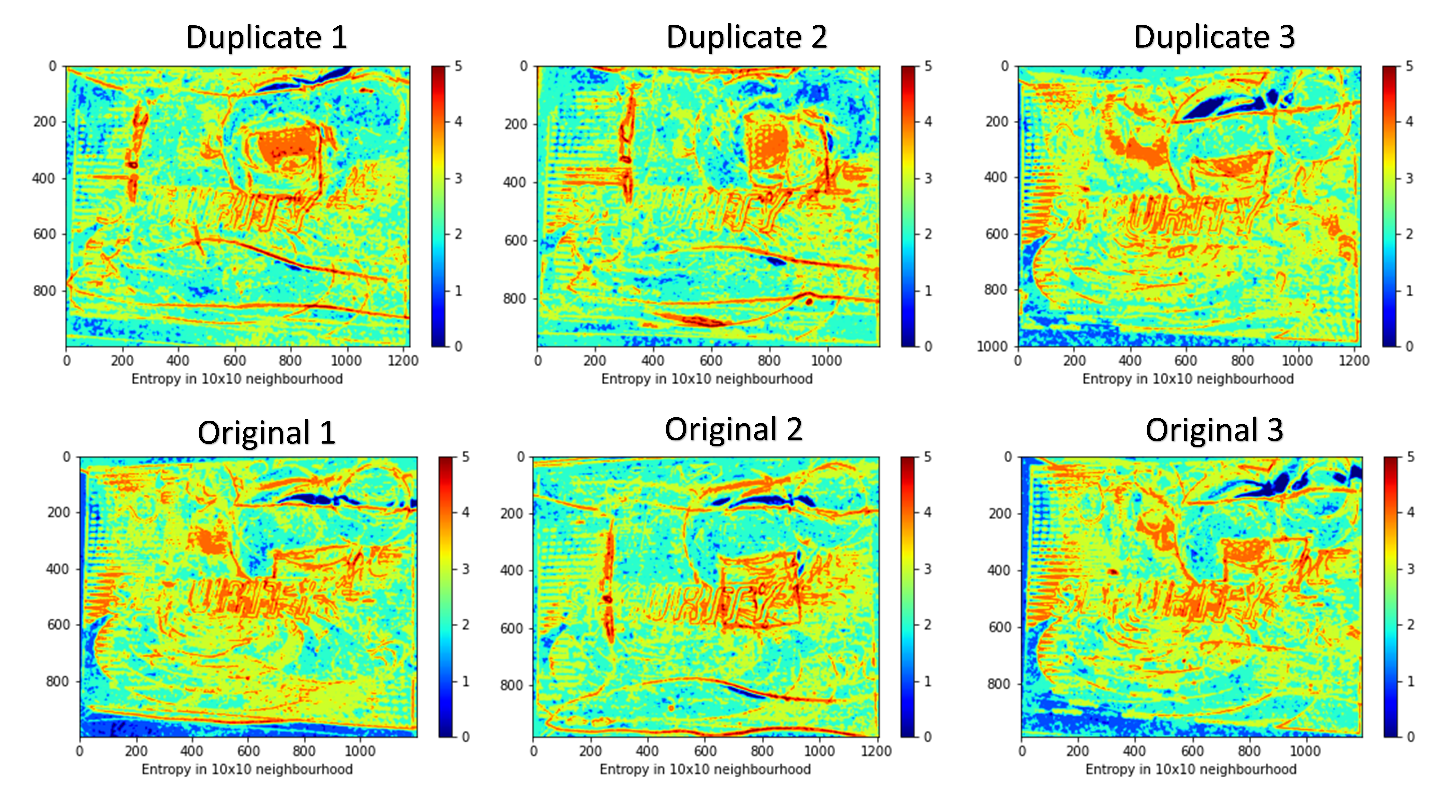


Figure S4. Entropy at 550 nm.


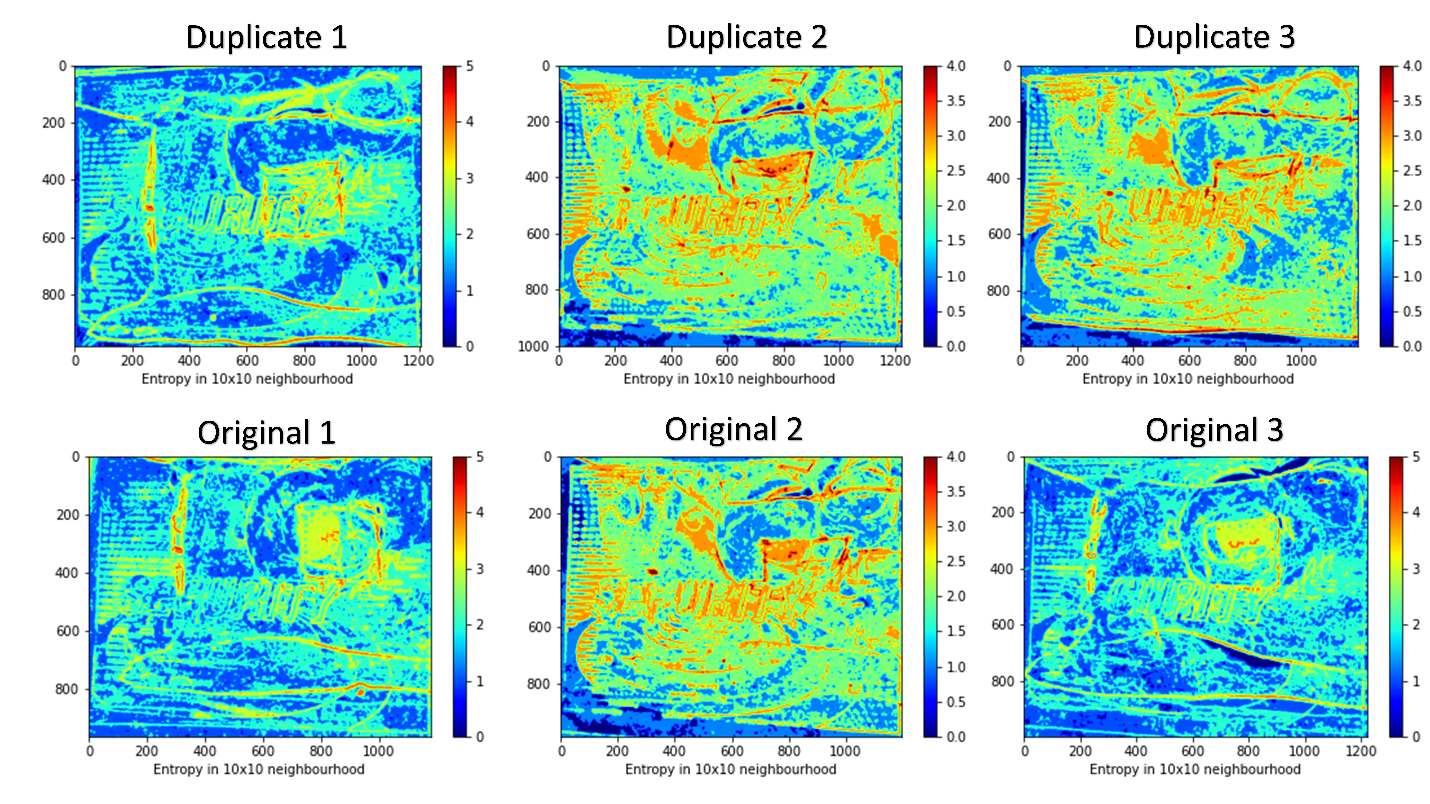


Figure S5. Entropy at 600 nm.


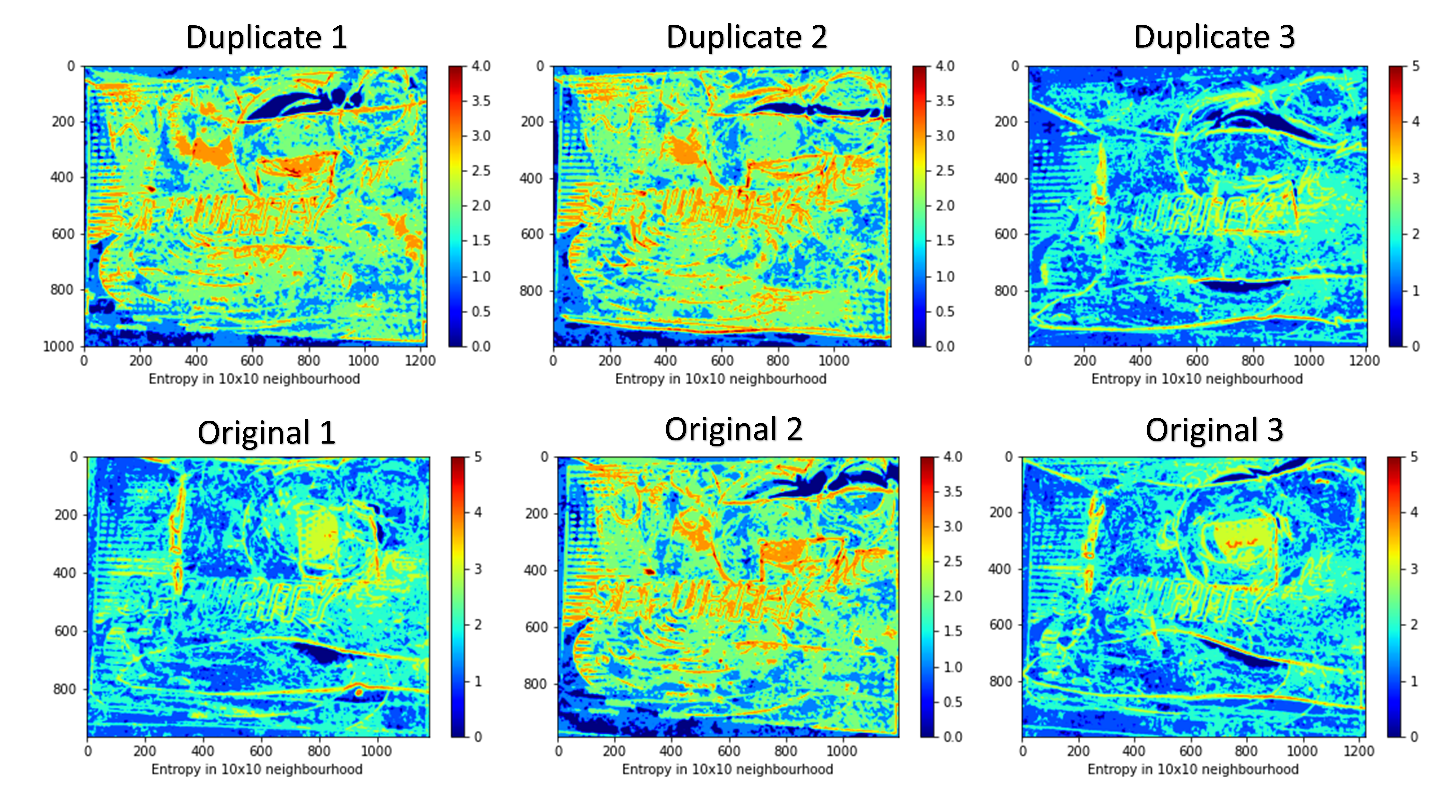


Figure S6. Entropy at 650 nm.


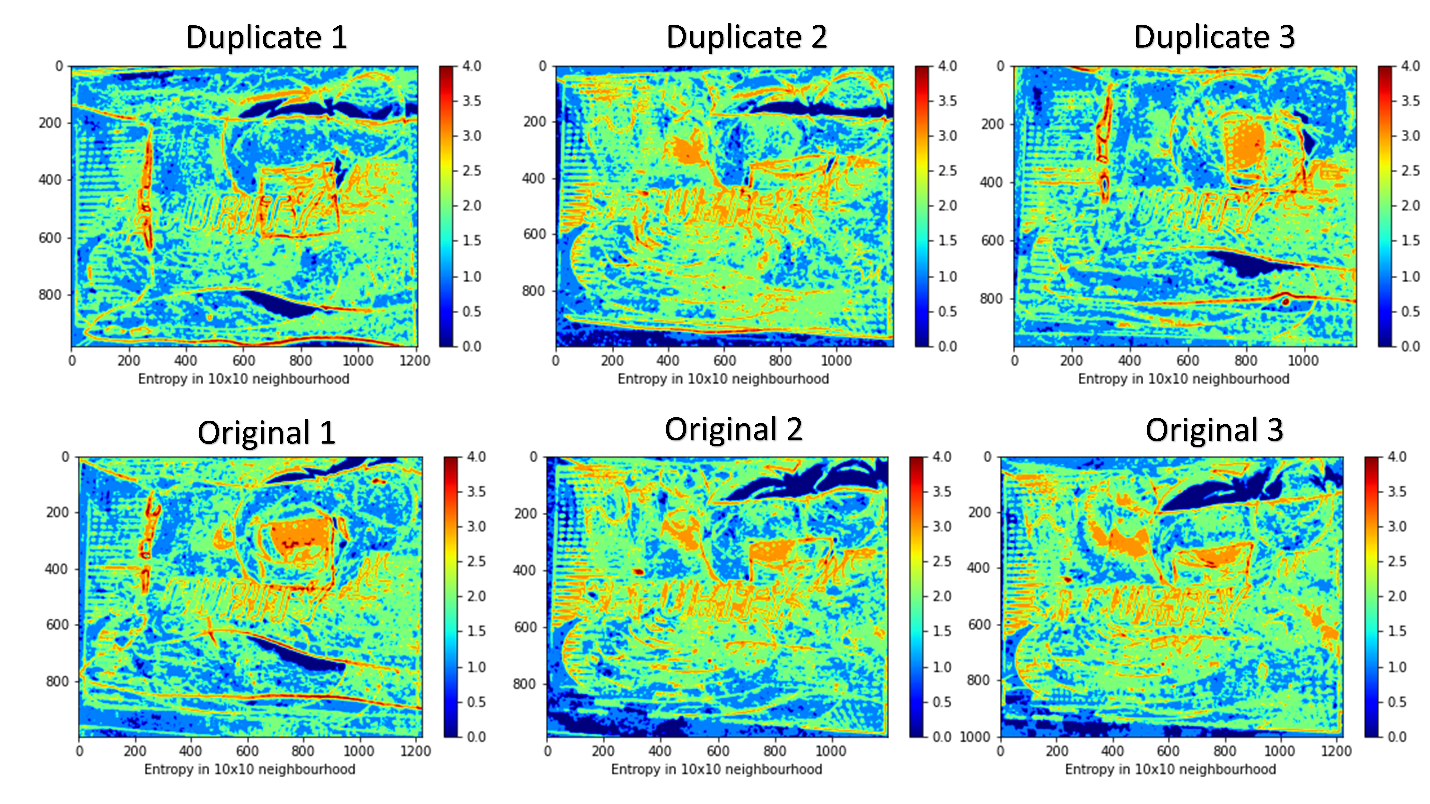


Figure S7. Entropy at 700 nm.

**3. Mean Gray Value and Reflection Analysis**

In this study, duplicate and original holograms are classified based on the MGV. Figure S8 shows the MGV of the eight samples (four original and four duplicate) used in this study.

Figure S8. MGV of the four duplicate and four original samples

As seen in the figure the MGVs of original and duplicate holograms are separated. Also, in this study, the reflection of the holograms was analyzed Figure S9 represents the reflectivity of the samples in the visible band.

Figure S9. Reflectance spectra of all the samples

It can be seen that after 580 nm there is a clear difference between the original samples and the duplicate samples.

**4. Visible Hyperspectral Imaging Algorithm**

The visible hyperspectral imaging (VIS-HSI) used in this study is calculated by using the images taken by a single-lens camera (Nikon D5200) combined with the visible hyperspectral algorithm (VIS-HSA). The wavelength range is from 380 nm to 780 nm, and the spectral resolution is up to 1 nm. The detailed instrument specifications used in this study are mentioned in Table 1.

|  | Specification | Resolution | Components | Bilateral |
| --- | --- | --- | --- | --- |
| Visible Light Camera | Nikon D5200 | 6000*4000 | CMOS | 380-780nm |
| Spectrometer | Ocean Optics QE65000 | 1mm | CCD | 200-1100nm |

Table S2. Instrument Specification.

The individual conversion formulas to convert the 24-colour patch image and 24 colour patch reflectance spectrum data to XYZ colour space are as follows

On the camera side: convert sRGB color gamut space to XYZ color gamut space

$\left[ \begin{aligned} X \\ Y \\ Z \end{aligned} \right]=[M_{A}]\left[ T \right]\left[ \begin{aligned} f\left( R_{sRGB} \right) \\ f\left( G_{sRGB} \right) \\ f(B_{sRGB}) \end{aligned} \right]\times100 , 0\leq{R_{sRGB} \atop\begin{aligned} G_{sRGB} \\ B_{sRGB} \end{aligned}} \leq1$ (S1)

其中

$\left[ T \right]=\left[ \begin{aligned} 0.4104 0.3576 0.1805 \\ 0.2126 0.7152 0.0722 \\ 0.0193 0.1192 0.9505 \end{aligned} \right]$ (S2)

$f\left( n \right)= \left\{ \begin{aligned} {(\frac{n+0.055}{1.055})}^{2.4}, n>0.04045 \\ \left( \frac{n}{12.92} \right), otherwise \end{aligned} \right.$ (S3)

$\left[ M_{A} \right]=\left[ \begin{aligned} \frac{X_{SW}}{X_{CW}} 0 0 \\ 0 \frac{Y_{SW}}{Y_{CW}} 0 \\ 0 0 \frac{Z_{SW}}{Z_{CW}} \end{aligned} \right]$ (S4)

On the spectrometer side: convert reflection spectral data to XYZ color gamut space

$X=k\int_{380nm}^{780nm} S\left( \lambda\right)R\left( \lambda\right)\bar{x}\left( \lambda\right)d\lambda$ (S5)

$Y=k\int_{380nm}^{780nm} S\left( \lambda\right)R\left( \lambda\right)\bar{y}\left( \lambda\right)d\lambda$ (S6)

$Z=k\int_{380nm}^{780nm} S\left( \lambda\right)R\left( \lambda\right)\bar{z}\left( \lambda\right)d\lambda$ (S7)

$k=100/\int_{380nm}^{780nm} S\left( \lambda\right)\bar{y}\left( \lambda\right)d\lambda$ (S8)

The nonlinear response of the camera can be corrected by a third-order equation, and the nonlinear response correction variable is defined as V_Non-linear_.

$V_{Non-linear}=\left[ X^{3} Y^{3} Z^{3} X^{2} Y^{2} Y^{2} X Y Z 1 \right]^{T}$ (S9)

In the dark current part of the camera, the dark current is usually a fixed value and does not change with the amount of incoming light, so a constant is given as the contribution of the dark current, and the dark current correction variable is defined as V_Dark_.

$V_{Dark}=[a]$ (S10)

Finally, VColor is used as the base, and multiplied by the nonlinear response correction of V_Non-linear_, and the result is standardized within the third order to avoid excessive correction, and finally V_Dark_ is added to obtain the variable matrix V.

$V_{Color}={[XYZ XY XZ YZ X Y Z]}^{T}$ (S11)

$V=\left[ X^{3} Y^{3} Z^{3} X^{2}Y X^{2}Z Y^{2}Z XY^{2} XZ^{2} YZ^{2} XYZ X^{2} Y^{2} Y^{2} XY XZ YZ X Y Z a \right]^{T}$ (S12)

Before using CIE DE2000 to calculate color difference, XYZ_Correct_ and XYZ_Spectrum_ must be converted from XYZ color space to lab color space. The conversion formula is as follows:

${L^{*} = 116f\left( \frac{Y}{Y_{n}} \right)-16 \atop\begin{aligned} a^{*} = 500\left[ f(\frac{X}{X_{n}})-f(\frac{Y}{Y_{n}}) \right] \\ b^{*} = 200\left[ f(\frac{Y}{Y_{n}})-f(\frac{Z}{Z_{n}}) \right] \end{aligned}}$ (S12)

$f\left( n \right)= \left\{ \begin{aligned} n^{\frac{1}{3}}, n>0.008856 \\ 7.787n+0.137931, otherwise \end{aligned} \right.$ (S13)

**5. Windows based Python Application**


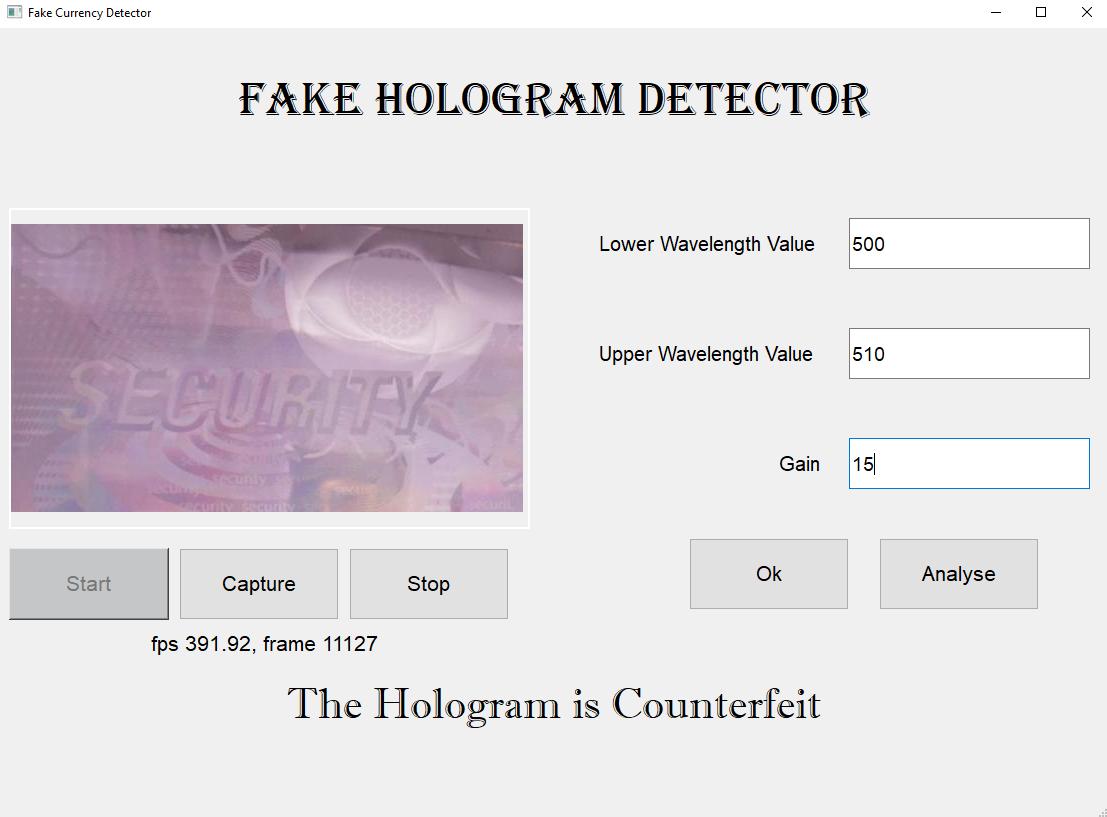


**Figure S10.** Fake hologram detector application was developed in this study.
